# Full-length versus truncated α-factor secretory signal sequences for expression of recombinant human insulin precursor in yeast *Pichia pastoris*: a comparison

**DOI:** 10.1186/s43141-023-00521-w

**Published:** 2023-05-22

**Authors:** Nuruliawaty Utami, Dini Nurdiani, Hariyatun Hariyatun, Eko Wahyu Putro, Fadillah Putri Patria, Wien Kusharyoto

**Affiliations:** 1 Research Center for Genetic Engineering, National Research and Innovation Agency (BRIN), Cibinong, Bogor 16911 Indonesia; 2 Research Center for Applied Microbiology, National Research and Innovation Agency (BRIN), Cibinong, Bogor 16911 Indonesia; 3 Laboratory Department, Indonesia International Institute for Life Sciences (i3L), Jakarta, Timur 13210 Indonesia

**Keywords:** Human insulin precursor, Secretory signal, Truncated α-factor, Full-length α-factor, *Pichia pastoris*, BMMY, BSMM

## Abstract

**Background:**

Human insulin was the first FDA-approved biopharmaceutical drug produced through recombinant DNA technology. The previous studies successfully expressed recombinant human insulin precursors (HIP) in *Pichia pastoris* truncated and full-length α-factor recombinant clones. The matting α-factor (Matα), a signal secretion, direct the HIP protein into the culture media. This study aimed to compare the HIP expression from full-length and truncated α-factor secretory signals clones that grown in two types of media, buffered methanol complex medium (BMMY) and methanol basal salt medium (BSMM).

**Results:**

ImageJ analysis of the HIP’s SDS-PAGE shows that the average HIP expression level of the recombinant *P. pastoris* truncated α-factor clone (CL4) was significantly higher compared to the full-length (HF7) when expressed in both media. Western blot analysis showed that the expressed protein was the HIP. The α-factor protein structure was predicted using the AlphaFold and visualized using UCSF ChimeraX to confirm the secretion ability for both clones.

**Conclusions:**

CL4 clone, which utilized a truncated α-factor in the *P. pastoris* HIP expression cassette, significantly expressed HIP 8.97 times (in BMMY) and 1.17 times (in BSMM) higher than HF7 clone, which used a full-length α-factor secretory signal. This research confirmed that deletion of some regions of the secretory signal sequence significantly improved the efficiency of HIP protein expression in *P. pastoris*.

## Background

The concept of recombinant protein from recombinant DNA harnessed the world’s most miniature manufacturing factories, living cells. Human insulin (HI) was the first medical biotechnology drug produced with this technology and was classified as the first biopharmaceutical approved by the FDA [1, 2]. Host cells can be used and selected for heterologous protein expressions, such as bacteria, yeast, insect cells, mammalian cells, transgenic animals, and transgenic plants [3–5]. The selection of host cells depends on protein requirements and character types (mass, purity, solubility, number of disulfide bonds, and model of post-translational modification), functional activity, and the necessity yields [6]. To date, yeast is the host cell of choice used for the production of recombinant human insulin because of its advantages over other cells [4]. *P. pastoris* could be a suitable expression system because it possesses a strong and tightly regulated *AOX1* promoter, grows rapidly, could achieve high cell densities, secretes appropriately folded protein, and provides post-transcriptional and post-translational modifications [7–10].

Naturally, proteins produced in eukaryotic cell have signal peptides that direct them to the export machinery so that the proteins can be secreted from the cell [11]. These properties became critical in the production of recombinant proteins that secrete the protein of interest into the external culture environment. During the development of recombinant human insulin research, several signal sequences were used to optimize the secretion process [12–16]. Only a few signal sequences are known for leading engineered protein in *P. pastoris*, including *P. pastoris* acid phosphatase (*PHO1*), *Saccharomyces cerevisiae SUC2* gene, and bovine β-casein signal sequences, which have been rarely used [17–20].

The most widely used secretion signal in *P. pastoris* is a mating α-factor signal sequence (Matα) derived from *S. cerevisiae* [7, 21, 22]. The 85 amino acid sequences of α-factor consisted of the 19 amino acids as pre-region followed by the 66 amino acids as a pro-region with three N-linked glycosylation sites at the amino acid numbers 23, 57, 67, and terminated by the Kex2 endo-proteinase cleavage site [23, 24]. The pre-peptide from the pre-region is considered crucial for interaction with signal recognition. The pre-region allegedly have a pivotal role in translocating the cargo protein to the endoplasmic reticulum (ER) co-translationally or post-translationally in yeast [25]. In contrast, the pro-region is thought to play an essential role in translocating the protein from the ER to the Golgi apparatus and ensuring the proper protein folding [23].

The processing of a Matα occurs in three steps. The first is elimination of the pre-signal (pre-region) by signal peptidase in the ER. The second step is the cleavage of the pro-peptide (pro-region) sequence between the Lys-Arg (KR) and the C-terminus by Kex2 endo-proteinase. Finally, the Ste13 protein cleaves the Glu-Ala (EA) repeats in the Golgi [22, 25]. The signal sequence becomes interesting that efforts have been made to increase the secretion efficiency of the protein of interest, especially in *P. pastoris*. Some strategies are applied to increase the secretion leader’s function through codon optimization, directed evolution, and spacer sequences [25–28].

The previous research conducted by Nurdiani et al. in [29] successfully secreted the HIP from *P. pastoris* transformants into the culture medium. The clones derived from *P. pastoris* used the construction of the insulin cassette with a truncated α-factor leader sequence (with a deletion in Matα: Δ30–43 and Matα: Δ57–70). The HIP expression cassette was regulated under the *AOX1* inducible promoter. The construct generated in the integrative vector pD902-IP consisted of the truncated α-factor secretory sequence equipped with the Kex2 endo-proteinase cleavage site (LEKR), spacer peptide (EEAEAEAEPK), followed by the insulin-B-chain (29 amino acids), a short C-peptide as a linker (DGK), and the last is continued by the insulin-A chain (21 amino acids).

While in our other study, *P. pastoris* recombinant strains expressing HIP using a full-length α-factor signal sequence upstream of the HIP expression cassette of pD902-IP have been established [30]. Therefore, in this study, the effect of the α-factor signal sequence modification on the expression of the HIP from both recombinant strains was compared.

## Methods

**Fig. 1:**
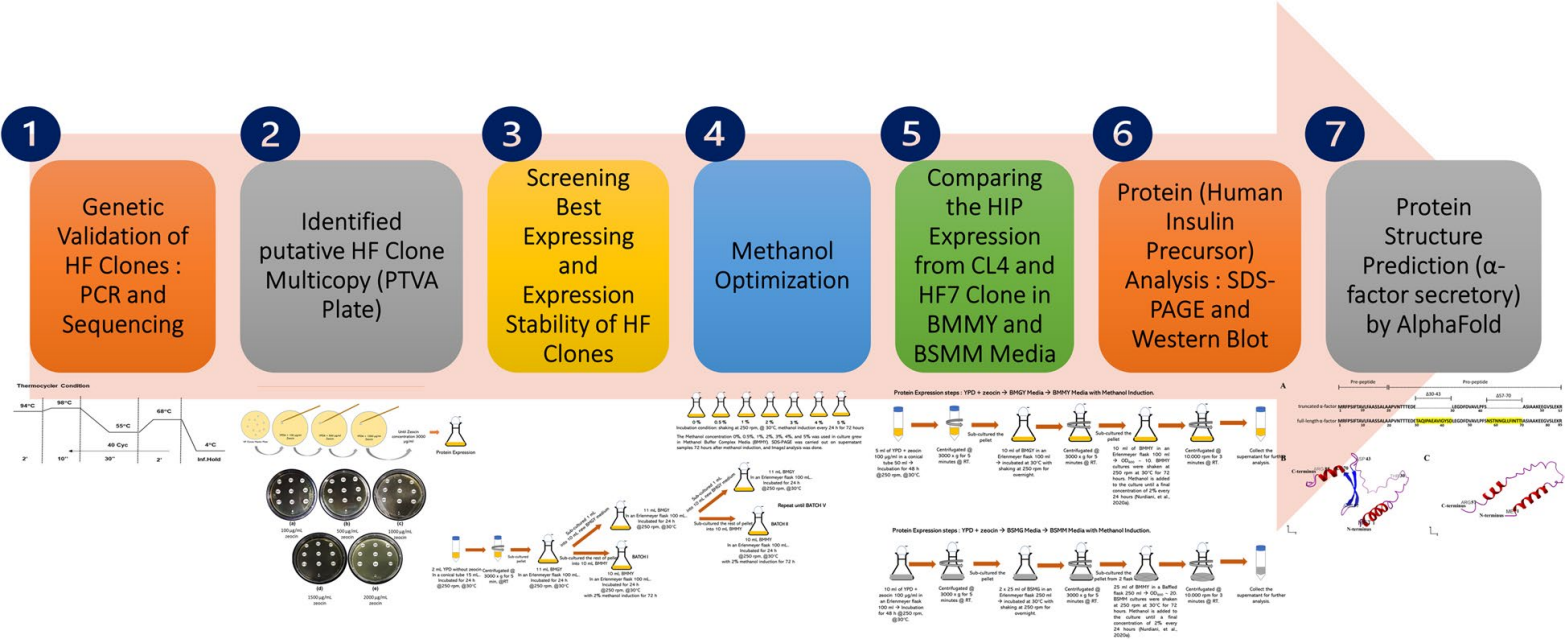
Flow diagram of the research procedures used in this study

In this study, as shown in Fig. 1, a series of steps were performed to compare the expression of human insulin precursor protein from full-length and truncated α-factor *P. pastoris* recombinant clones. The seven stages of the research consisted of genetic validation of full-length (HF) clones by PCR and sequencing, identified HF clone multicopy by PTVA plate, screening best expressing and expression stability clone analysis, methanol optimization, flask scale fermentation process to compare HIP expression from full-length and truncated α-factor clones in two kinds of media, protein analysis by SDS-PAGE and Western blot, and protein (α-factor secretory signal) structure prediction by AlphaFold.

### Strains

Two recombinant *P. pastoris* clones described from the previous study [29, 30] that express the HIP protein into the culture media were used. The CL4 clone from *P. pastoris* X-33 (Invitrogen K1740-01, Carlsbad, CA, USA) used a truncated α-factor leader sequence for the HIP expression cassette (pD902-IP) [29]. The CL4 is one of the best expressing clones [31], and its expression stability has been confirmed [32]. The second clone is a *P. pastoris* recombinant human insulin full-length α-factor (HF), which is derived from the subclone of the full-length α-factor into the pD902-IP cassette (pD902-IP-full-length-α-factor) was established in our previous work [30].

### Molecular verification of HF transformants

The full-length α-factor was successfully subcloned into the pD902-IP cassette and was used to transform the *P. pastoris* X-33 strain [30]. The integration of insulin-encoding genes into the genome was analyzed by PCR. Prior to PCR, the nine selected recombinant yeast (HF) genomes were extracted using the YeaStar Genomic DNA Kit D2002 (Zymo Research Corp., Irvine, USA) and were used as templates. Confirmation of the methanol utilization (Mut) phenotype using an *AOX1*-specific primer set listed in the EasySelect™ Pichia Expression Manual [33] is as follows: *AOX1*F (5-GAC TGG TTC CAA TTG ACA AGC-3′) and *AOX1*R (5-GCA AAT GGC ATT CTG ACA TCC-3′). The PCR with Toyobo, KOD-401, (KOD-Plus-Neo, Osaka, Japan) kit used was the pre-denaturation condition at 94 °C for 2 min followed by 40 cycles of denaturation at 98 °C for 10 s, annealing at 55 °C for 30 s, elongation at 68 °C for 2 min, then terminated at 4 °C for infinite hold. The total reaction volume for each DNA genome template was 50 μL, with a final concentration of KOD-Plus-Neo enzyme for each reaction was 1.0 U/50 μL.

The 10 μL PCR product was electrophoresed in a 1% agarose gel at 100 V for 30 min. The gel was visualized with Syngene G:BOX Gel Documentation System (Sygene, MD, USA) after 15 min of immersion in 0.5 μg/mL EtBr Sigma E1510 solution (Sigma-Aldrich, Burlington, MA, USA). Sequencing analysis of the amplicon (40 μL PCR product) was performed by 1st BASE DNA Sequencing (Selangor, Malaysia). Multiple sequence alignment analysis was performed using DNAMan1 (Lynnon Biosoft, CA, USA).

### Post-transformational vector amplification (PTVA) plate method

**Fig. 2:**
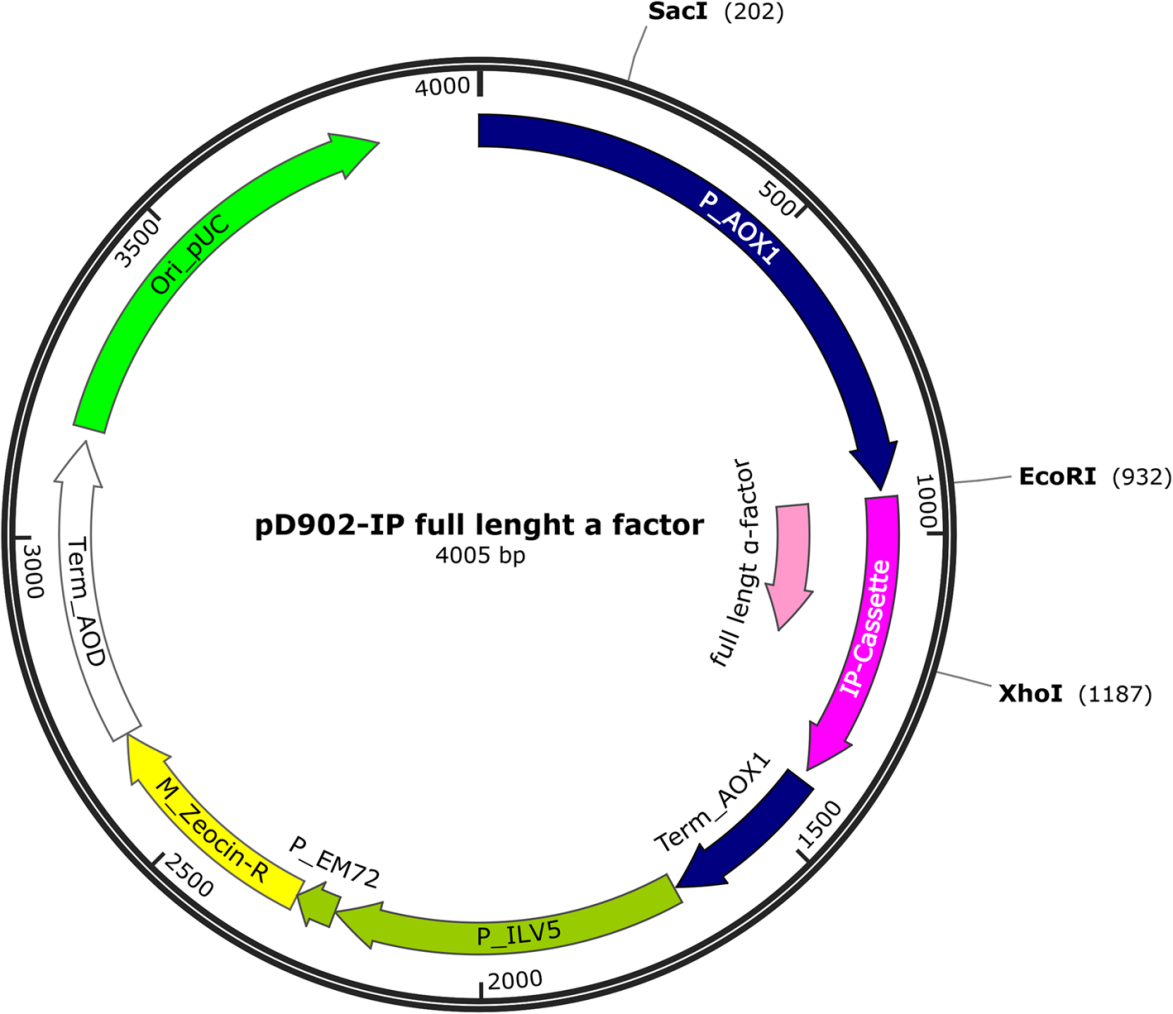
pD902-IP-full-length-α factor expression cassette map

The PTVA plate method was used to identify suspected multicopy HF clones. This method uses the antibiotic zeocin as a selective agent (selectable marker) when recombinant clones become resistant to it because the zeocin-resistant gene has been integrated into the expression cassette (Fig. 2) transformed into the host cell. Nine HF clones (HF1-3, HF5-10) and one wild-type *P. pastoris* X-33 were grown on a YPD agar plate [1% yeast extract (w/v), 2% peptone (w/v), 2% D-glucose (w/v), and 2% bacto-agar (w/v)] with graded concentrations of Zeocin ThermoFisher R25001 (Invitrogen ThermoFisher Scientific, MA, USA) ranging from 100 to 2000 μg/mL. Colonies on the plate with the lowest zeocin concentration plate were incubated at 30 °C for 5 days. On the fifth day, all colonies were plated on new agar plates containing increasing concentrations of zeocin in YPD and incubated at 30 °C for 5 days. This plating was repeated until the zeocin concentration in the YPD plate reached 2000 μg/mL [34, 35].

### HIP expression of HF PTVA clones

Each clone from the PTVA results that could grow at the highest concentration of zeocin was cultured on 2 mL of YPD medium [1% yeast extract (w/v), 2% peptone (w/v), and 2% D-glucose (w/v)] with the addition of 100 μg/mL zeocin. The culture was incubated for 48 h at 30 °C with shaking at 250 rpm. The cells were then harvested by centrifugation at 3000 × *g* for 5 min at room temperature. The pellets were transferred to 10 mL of buffered glycerol complex medium (BMGY) in a 100-mL flask [1% yeast extract (w/v), 2% peptone (w/v), 100 mM potassium phosphate pH 6.0, 1.34% YNB (w/v), 4 × 10^−5^% biotin (w/v), and 1% glycerol (v/v)] and incubated overnight at 30 °C with shaking at 250 rpm. The culture was then centrifugated at 3000 × *g* for 5 min, and the cells were transferred to 10 mL buffered methanol complex medium (BMMY) [1% yeast extract (w/v), 2% peptone (w/v), 100 mM potassium phosphate pH 6.0, 1.34% YNB (w/v), 4 × 10^−5^% biotin (w/v), and 2% methanol (v/v)] to achieve OD_600_ ~10. BMMY cultures were shaken at 250 rpm at 30 °C for 72 h, accompanied by methanol induction added to the culture until a final concentration of 2% every 24 h. After 72 h of methanol induction, the cultures were harvested with centrifugation at 10,000 rpm for 3 min at room temperature. The supernatant was stored at – 20 °C for further analysis, and the SDS-PAGE [36] was performed. ImageJ software examined the protein expression results for semi-quantitatively analysis.

### Screening for the best expression of HF PTVA clone and clone stability

A total of five PTVA clones with the highest protein expression level were cultured in the BMMY media (preceded by YPD and BMGY pre-culture) by induction of a final concentration of 2% methanol every 24 h for 72 h at 250 rpm at 30 °C. ImageJ analysis of the SDS-PAGE results is used to select clones with the highest HIP expression.

Expression stability of the best expressing HF PTVA clone was preceded by clone growth evaluation on YPD and BMGY media to define the clone generation. HIP expression was examined without zeocin selection in the culture and induction media. One colony of the best expressing HF PTVA *P. pastoris* recombinant clone was inoculated into 2 mL of YPD medium and agitated at 250 rpm and 30 ℃ for 24 h. The culture was centrifuged at room temperature for 5 min at 3000 × *g* to collect the cells. The cell pellet was then transferred to 11 mL of BMGY medium in a 100-mL flask and cultured for 24 h at 30 °C and 250 rpm in a shaking incubator. After 24 h of culture, 1 mL aliquots were taken to inoculate the BMGY culture medium for the next generation. The remaining cells were then collected by centrifugation at 3000 × *g* for 5 min at room temperature. The cell pellet was resuspended in 10 mL BMMY medium in a 100-mL flask to obtain an OD_600_ ~10.

Methanol induction was performed every 24 h to a final concentration of 2%. After 72 h of methanol induction, the supernatant was collected in a separate tube (centrifugated at 6000 × *g* for 3 min at room temperature) and stored at – 20 °C until protein analysis. The HIP expression cycle was repeated using 1 mL of the previous BMGY culture up to the fifth batch, with each batch having 12.3 generation differences. HIP expressions were analyzed by SDS-PAGE and genetic stability was evaluated by PCR genome using the *AOX1* primer in each batch.

### Methanol concentration optimization

After obtaining the best-expressing HF PTVA clones, the optimization of the methanol concentration in the expression process was done to determine what percentage of the optimal methanol should be used. The final concentrations of 0, 0.5, 1, 2, 3, 4, and 5% methanol were used in the culture grown in the BMMY expression medium. SDS-PAGE was applied to supernatant samples 72 h after methanol induction, and ImageJ analysis was performed.

### Expression of the HF vs. CL4 clone in BMMY and BSMM media

The selected HF clones were known from screening, and their expression was compared with that of the CL4 clones. Each HF, CL4, and wild-type (WT) clone was expressed in triplicate in two media types: BMMY [33] and methanol basal salt medium (BSMM), as previously described by Wu et al. in [37]. Clone expression on BMMY and BSMM media was preceded by inoculation in a YPD medium with zeocin 100 μg/mL (except for the WT clone). All cultures were incubated for 48 h at 250 rpm shaking at 30 °C. The pellet from a 2 mL of YPD medium (in a 15-mL polypropylene tube) was harvested with centrifugation (3000 × *g* for 5 min at room temperature). Cells from each clone were subcultured into 10 mL (in 100-mL Erlenmeyer flask) of BMGY and incubated at 250 rpm at 30 °C for 24 h. Each culture was centrifuged at 3000 × *g* for 5 min, and the pellet was grown into 10 mL BMMY in a 100-mL flask with 2% (v/v) methanol added. The BMMY culture was shaken at 250 rpm at 30 °C with 2% methanol (v/v) added. 50% methanol solution was added every 24 h to a final concentration of 2% to maintain the induction. After three days, the supernatant was collected by centrifugation at 10,000 rpm and 4 ℃ for 3 min and then analyzed by SDS-PAGE.

For expression in BSMM, the 10-mL YPD culture for each clone (in 100-mL Erlenmeyer flasks) was centrifuged (3000 × *g* for 5 min at room temperature) and then subcultured into 2 × 25 mL of glycerol basal salt medium (BSMG) (each one in 250-mL Erlenmeyer flasks). BSMG consisted of ½ BSM, 40 g/L glycerol, 4.35 mL/L biotin 500 × , 4.35 mL/L PTM1 trace salts with 12.5% ammonia solution until the pH of the medium reached five, and sterilized water was added to the desired volume. The composition of the BSM medium was 0.465 g/L CaSO_4_ × H_2_O, 9.1 g/L K_2_SO_4_, 7.45 g/L MgSO_4_ × 2H_2_O, 2.65 g/L KOH, 13.35 mL/L 85% H_3_PO_4_. The PTM1 trace salts include 6.0 g/L CuSO_4_ × 5H_2_O, 0.08 g/L NaI, 3.0 g/L MnSO_4_ × H_2_O, 0.2 g/L Na_2_MoO_4_ × 2H_2_O, 0.02 g/L boric acid, 0.5 g/L CoCl_2_ × 6H_2_O, 20 g/L ZnCl_2_, 65 g/L FeSO_4_ × 7H_2_O, 0.2 g/L biotin, and 5.0 mL/L H_2_SO_4_ [37]. The BSMG culture was incubated at 30 °C with shaking at 250 rpm for 24 h.

In addition, the cells from each culture were harvested by centrifugation at 3000 × *g* for 5 min at room temperature. Cells from 2 × 25 mL BSMG were combined into one and then subcultured into 25 mL BSMM in a 250-mL baffled flask, and the OD_600_ reached ~20. The BSMM medium was consisted of ½ BSM, 4.35 mL/L biotin 500 × , 4.35 mL/L PTM1 trace salts, 50% methanol solution to a final concentration of 2% (v/v), 12.5% ammonia solution to adjust the pH of the medium to five, and sterilized water to the desired volume. The culture was grown by shaking at 250 rpm at 30 °C for 72 h. Methanol induction was applied to each culture every 24 h.

### Protein analysis with SDS-PAGE and Western blot

For the 72 h BMMY and BSMM supernatants, HIP was assessed by SDS-PAGE. However, Western blot analysis was only performed on the BSMM supernatant. SDS-PAGE for BMMY supernatant was performed on a 15% polyacrylamide gel using a Tricine buffer system [36]. Approximately 50 μL of supernatant from each clone was mixed with an equal volume of Bio-Rad Tricine sample buffer #1610739 (Bio-Rad, CA, USA). Samples were incubated in a dry bath at 70 °C for 10 min and loaded onto the gel (20 μL per well). The gel was electrophoresed at 132 V, 400 A, for 120 min using a Tricine running buffer. The separated polypeptides were visualized using Coomassie Brilliant Blue (CBB) R-250 staining solution #1610436 (Bio-Rad, CA, USA). The gel was destained with 15 mL destaining solution I [53% water (v/v), 40% methanol (v/v), 7% acetic acid (v/v)] for 30 min, followed by 20 mL destaining solution II [88% water (v/v), 5% methanol (v/v), 7% acetic acid (v/v)] for 2 h.

Meanwhile, the BSMM supernatant was electrophoresed using the precast gel of GenScript SurePAGE, Bis–Tris, 10 cm × 8 cm gels gradient (4–20%) M00657 (GenScript Biotech, Nanjing, Jiangsu, China) in GenScript running buffer Tris-MOPS-SDS 1 × M00138 (GenScript Biotech, Nanjing, Jiangsu, China) at 200 V for 35 min. Western blot analysis was performed using the modified method of the Okita et al. [38]. Up to 250 μL of CL4 and HF culture supernatant was collected 72 h after methanol induction and lyophilized overnight. Freeze drying was performed to concentrate the HIP from the supernatant and improve the protein band’s visibility in SDS-PAGE and Western blot procedures. Lyophilized HIP from each clone was diluted in 50 μL nuclease-free water and used for SDS-PAGE and Western blot. The blot was performed using a wet blotting system. A PVDF membrane Immun-Blot® PVDF Membrane, Precut, 7 × 8.4 cm #1620174 (Bio-Rad, CA, USA) was used, run in an electrotransfer buffer at 90 V for 120 min, and the transfer container was surrounded by ice pack gel (maintaining a chill environment). The human insulin USP standard Sigma 1342106 (Sigma-Aldrich, Singapore) was used as a reference for both SDS-PAGE and Western blot analysis. The primary antibody Insulin/Proinsulin monoclonal antibody, ThermoFisher Scientific, Invitrogen/D3E7, 5B6/6 (Invitrogen, Carlsbad, CA, USA) and blocking buffer at 1:1000 (10 μL antibody in 10 mL blocking buffer) were used overnight with shaking at 4 °C. The secondary antibody Anti-mouse IgG (H + L) AP Conjugate, Promega #S3721 (Promega Corporation, Madison, WI, USA) and TBST (a mixture of tris-buffered saline (TBS) and Tween 20) at 1:1250 were applied. Incubation was performed for 1 h at room temperature with shaking. Approximately 5 mL of NBT/BCIP Bio-Rad #1706432 (Bio-Rad, CA, USA) was applied to visualize the sandwich of complex protein detection in a dark, followed by incubation at room temperature for 5–15 min. The color visualization reaction was stopped with distilled water to record the result.

### Protein structure prediction

To observe the difference between the full-length and truncated α-factor secretory signal, the protein structure of both secretory signals used in the pD902-IP expression cassette has been predicted. The computational method AlphaFold using DeepMind’s Colab notebook (https://colab.research.google.com/github/deepmind/alphafold/blob/main/notebooks/AlphaFold.ipynb, accessed on September 8, 2022) simplified version of AlphaFold v2.3.1. [39, 40] was used to predict the three-dimensional structure of the Matα secretory protein based on its amino acid sequence. After prediction, the molecular graphic of the protein structure was visualized using UCSF ChimeraX 1.4 [41, 42].

### Data and statistical analysis

HIP expression was analyzed using ImageJ 1.53t (Wayne Rasband and contributors National Institutes of Health, USA, https://imagej.nih.gov/ij) [43]. Statistical data analysis was performed using IBM SPSS Statistics for Windows, version 26.0. Armonk, NY: IBM Corp. [44].

### Characterization of recombinant HF clones

**Fig. 3:**
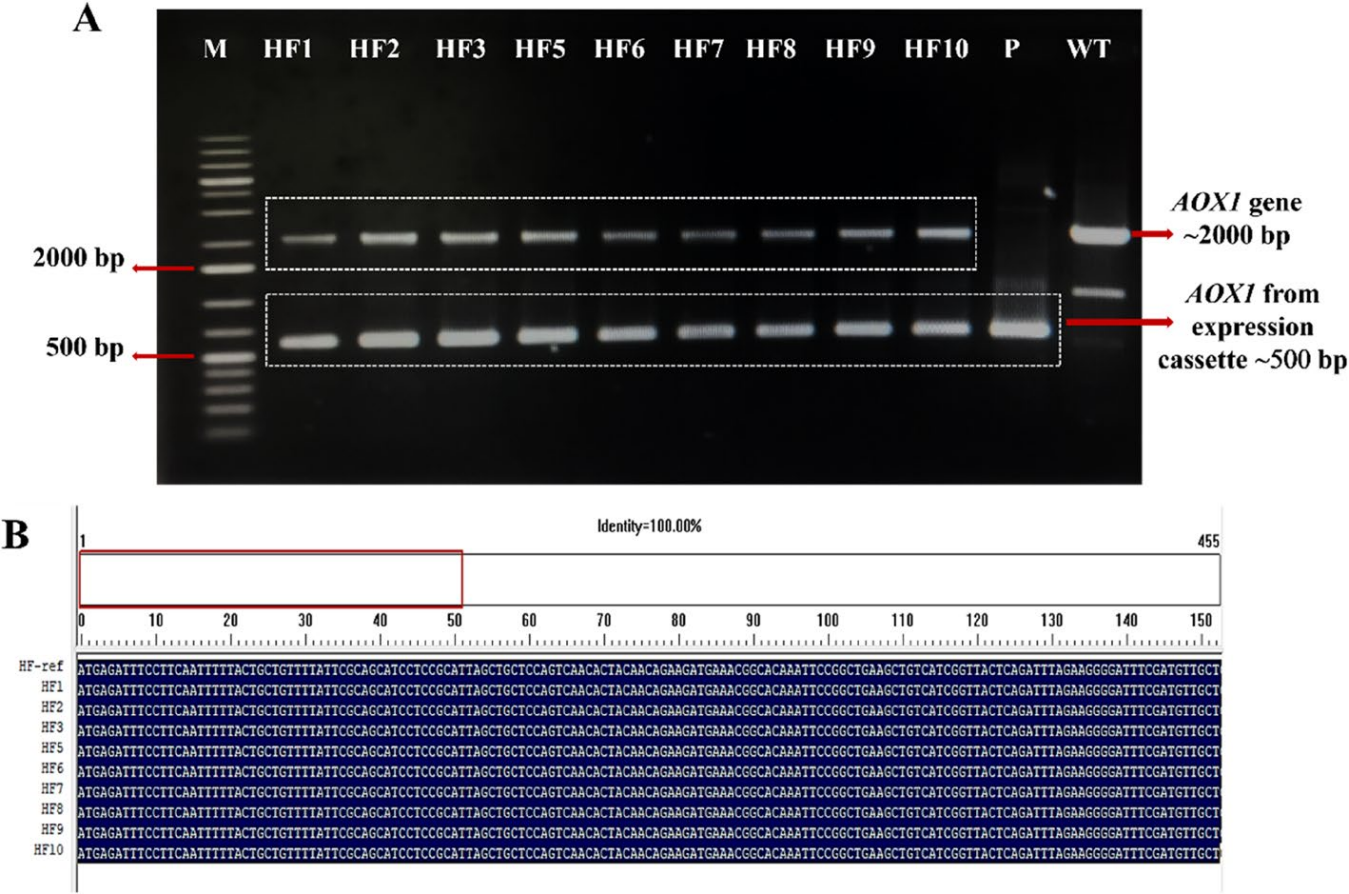
Genetic validation of nine recombinant *P. pastoris* (HF) clones. **A** PCR validation method. M = 1 kb DNA ladder; Lane 2–10 = amplicon from nine HF genomes, P = pD902-IP-full-length-α-factor plasmid as a template; WT = wild-type genome. **B** Multiple sequence alignment of nine recombinant HF clones

Genetic validation by PCR and sequencing of nine HF clones (HF1, HF2, HF3, HF5, HF6, HF7, HF8, HF9, and HF10) confirmed that the pD902-IP-full-length α-factor cassette was integrated into the *P. pastoris* genome. As shown in Fig. 3A, the *AOX1* PCR of HF clones resulted in two bands at ~ 500 and ~ 2000 bp. The band at ~ 500 bp band indicated the *AOX1* sequence from the IP cassette (pD902-IP-full-length α-factor), while the ~ 2000 bp band indicated that the *AOX1* gene originated from the yeast’s genome, as the ~ 500 bp band was not found in the wild-type (WT) genome template. Sequence analysis was performed to confirm the *AOX1* sequences of nine HF clones. The ClustalW multiple sequence alignment analysis of nine HF clones resulted in 100% identity of *AOX1* sequences, as shown in Fig. 3B.

**Fig. 4:**
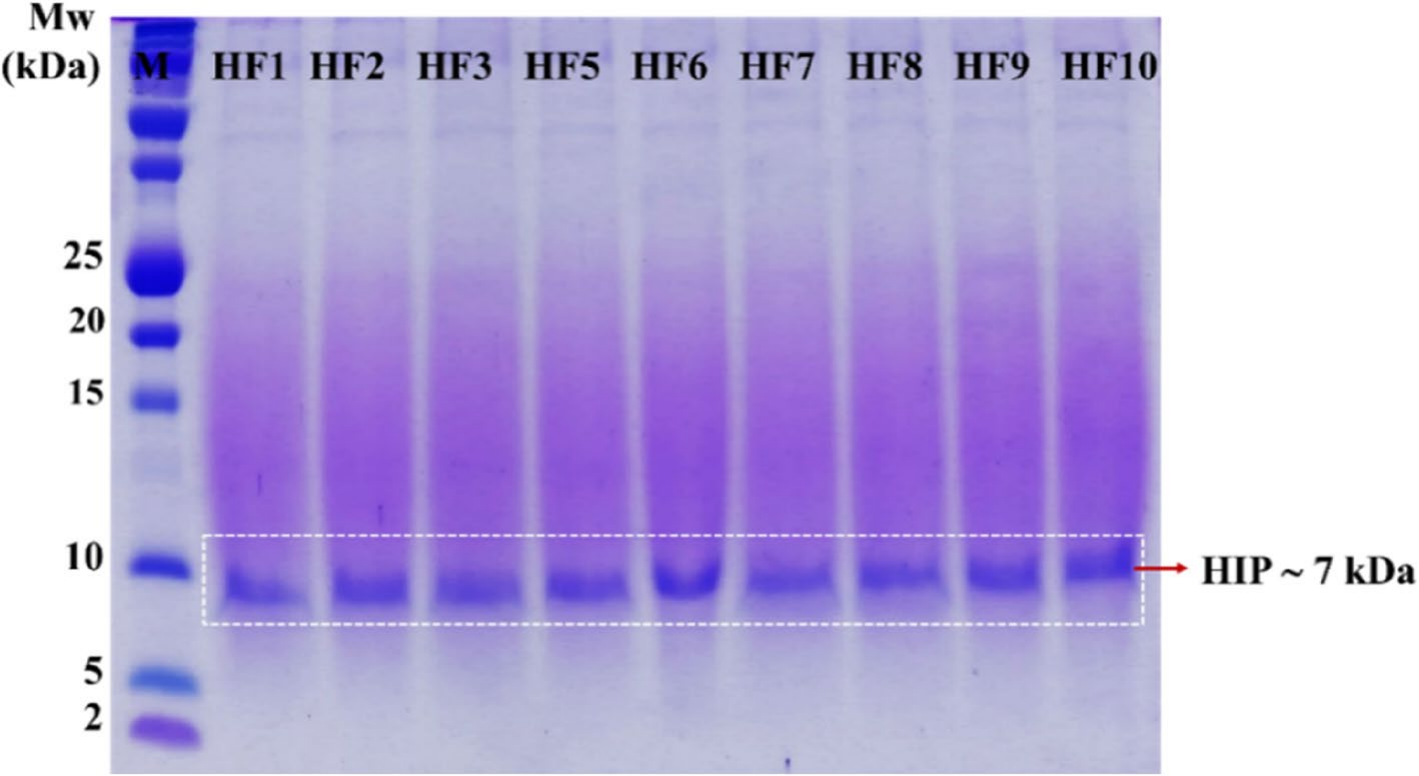
HIP expression of nine HF clones (putative multi-copy) 72-h supernatant with 2% methanol induction. M = protein marker (Precision plus dual Xtra, Bio-Rad, CA, USA). Lane 2–10 = HF clones

After the genetic validation of all HF clones was confirmed, the PTVA was conducted to identify the putative multi-copy HF clones. Similar to the CL4, exposure of the nine HF clones to zeocin at increasing concentrations on a YPD agar plate showed that all HF clones could be recovered up to 2000 μg/mL zeocin concentration. Subsequently, the HIP expression of the nine HF clones was confirmed in a BMMY with a final concentration of 2% methanol induction. The SDS-PAGE analysis showed that the nine clones secreted the HIP protein into the culture supernatant with a predicted molecular weight of ~ 7 kDa, as shown in Fig. 4.

**Fig. 5:**
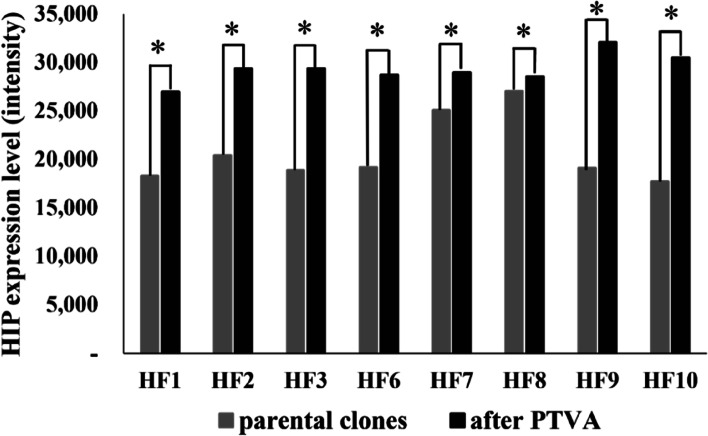
The HIP expression intensity of eight parental HF clones compared to their derivative PTVA clones. Asterisks (*) indicate significantly different comparisons according to the Wilcoxon signed ranks test (*p* value < 0.05)

In addition, the SDS-PAGE results were examined using ImageJ to evaluate the level of HIP expression from HF PTVA clones, as shown in Fig. 5. The intensity level captured by ImageJ describes the level of HIP expression. The expression of the parental clones and the PTVA clones derived from them was compared. The total HIP expression level of eight HF PTVA clones was significantly higher than their parental clones.

### Best expression and expression stability HF PTVA clones

**Fig. 6:**
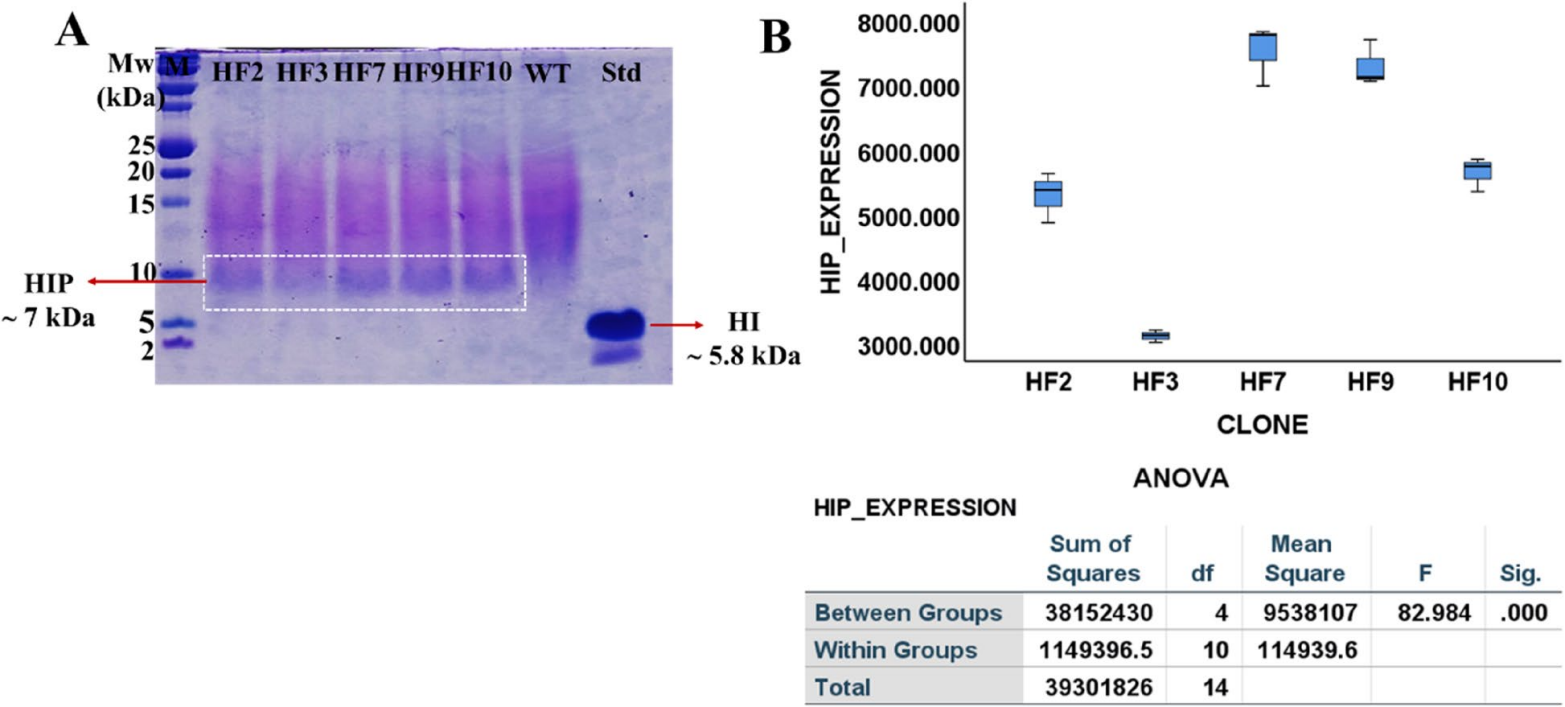
**A** HIP expression of five best expressing clones. **B** ANOVA analysis of five HIP expressions, *p* value  < 0.05 indicates that means in all HF clones are significantly different. The HIP expression stability of the HF7 clone was determined for five batches, each with 12.3 generations, based on growth evaluation on YPD and BMGY media

It shows that the five clones with the highest HIP expression level are HF2, HF3, HF7, HF9, and HF10. The five clones were re-expressed in a BMMY medium for the following observation to determine the best expressing clone. From the ImageJ analysis of HIP expression shown in Fig. 6, the HF7 clone is selected as the best expressing clone for HIP production in *P. pastoris* using a full-length α-secretory signal sequence. HF7 is used for subsequent comparative studies with CL4.

**Fig. 7:**
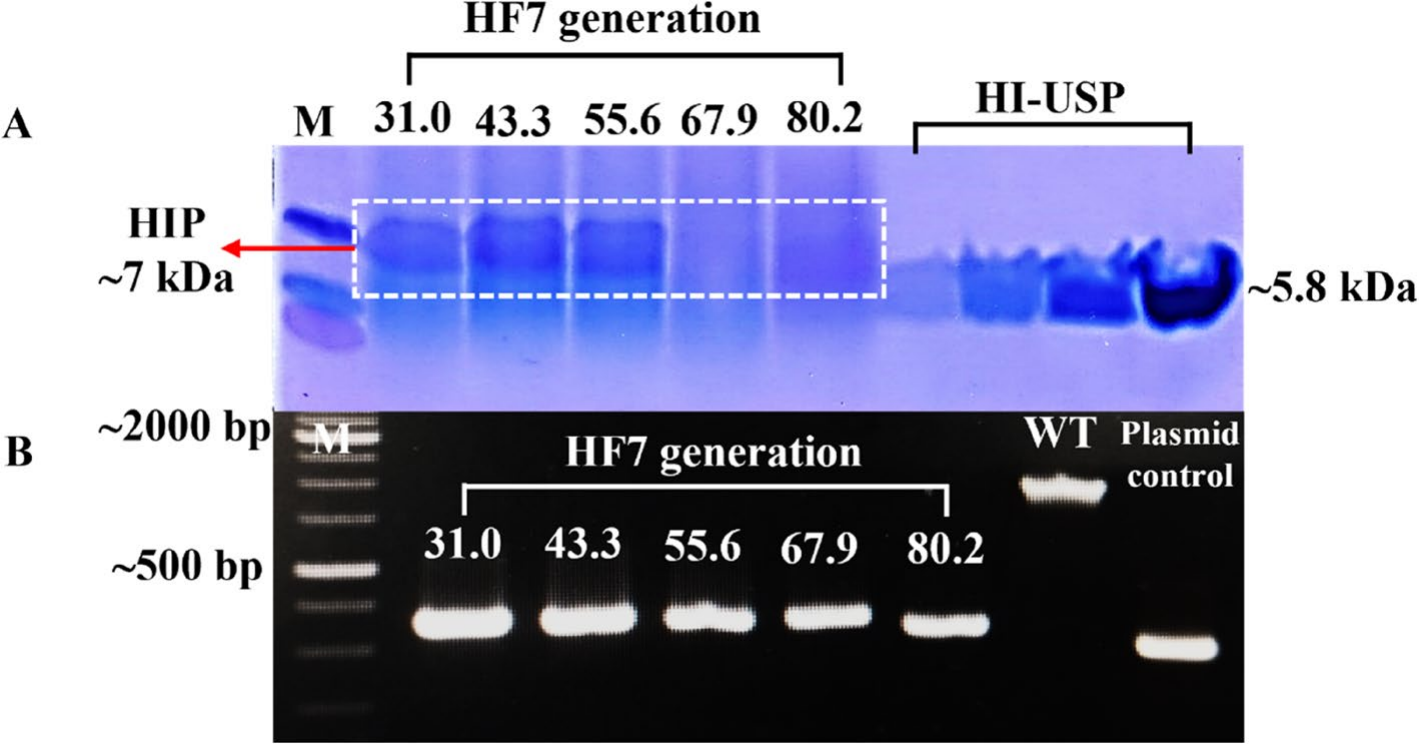
**A** Stability of five batch HF7 HIP expressions on BMMY medium. **B** Genetic stability assessment by PCR of five batch HF7 clone generations. WT = *P. pastoris* X33 (wild-type). Plasmid control = pD902-IP-full-length-α factor expression cassette

Figure 7 shows the HF7 HIP expression and PCR genome result from generation 30.1 to 80.2.

The expression stability assay shows that the HF7 clone still maintains the cassette of expression until generation 80.2, but the HIP only expresses until generation 55.6 without zeocin administration.

### Methanol concentration for the HIP secretion of HF7 and CL4 clones

**Fig. 8:**
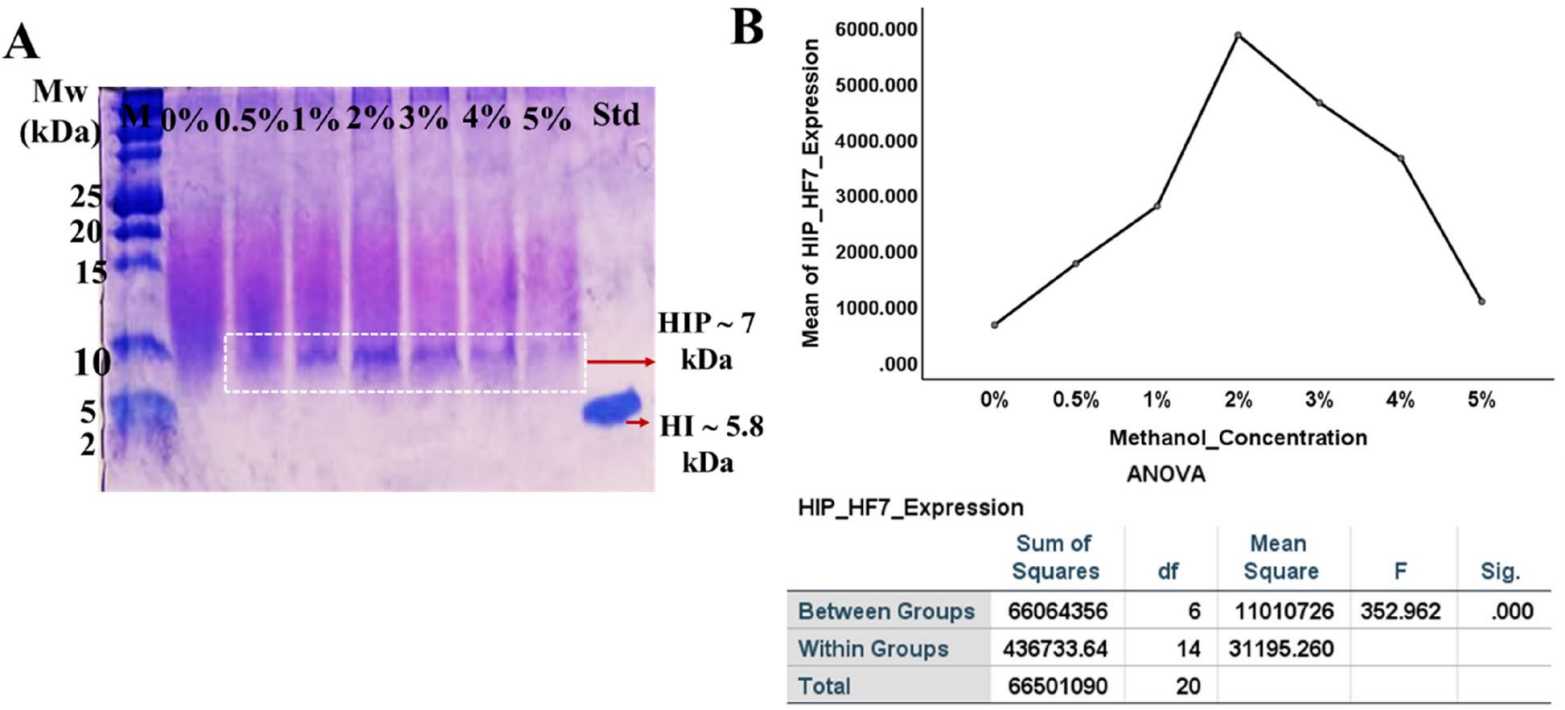
SDS-PAGE of methanol optimization. **A** The 72-h culture supernatant of HF7 clone with different concentrations of methanol induction. Lanes 2–8 are HIP expressions at 0, 0.5, 1, 2, 3, 4, and 5% methanol, respectively. HI = human insulin standard (1.82 mg/mL). **B** ANOVA analysis of HIP expressions at different methanol concentrations, *p *value < 0.05 indicates that the means in all groups are significantly different

Since the recombinant clones express the protein of interest under the regulation of the *AOX1* promoter, it is essential to determine the optimal methanol concentration to induce expression processes and thereby increase protein secretion. Therefore, some methanol concentration (0–5%) induction on HF7 clones was investigated to determine the optimal condition for HIP secretion. After 72 h of methanol induction, the culture supernatant was harvested and verified by SDS-PAGE, as shown in Fig. 8A. Methanol induction at 0% did not result in HIP expression, but 1, 2, 3, 4, and 5% methanol induction did. On the other hand, Fig. 8B shows the ImageJ intensity measurement of HIP expression.

Figure 8B shows that methanol induction at a final concentration of 2% resulted in the highest HIP production compared to other concentrations. Based on these results, methanol induction at a final concentration of 2% was used to compare the insulin precursor expression of HF7 and CL4.

### The HIP expression of CL4 vs. HF7 recombinant clones in BMMY and BSMM media

**Fig. 9:**
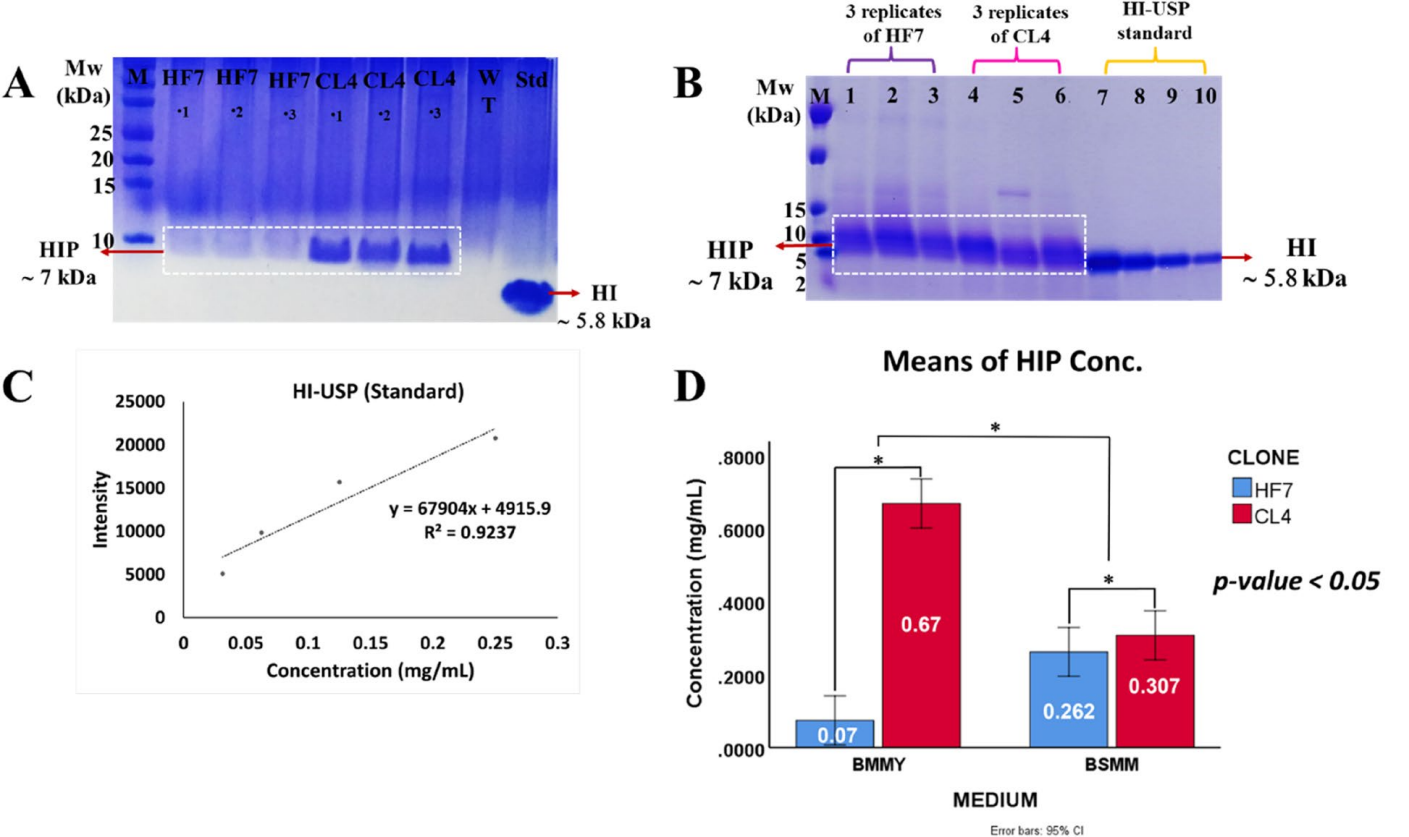
HIP expression of HF7 versus CL4 recombinant clones. **A** Comparison of expression in BMMY medium. HF7 and CL4 were replicated three times in one batch. M = Precision plus protein™ dual-color standard (Bio-Rad). **B** A comparison of the HIP expression in the BSMM medium. Lane 1–3 = three replicates of HF7, lane 4–6 = three replicates of CL4, lane 7–10 = USP human insulin standard (0.25, 0.125, 0.0625, 0.03125 mg/mL, respectively). **C** HI-USP standard regression analysis. **D** Two-way ANOVA analysis of HIP’s HF7 and CL4 concentration in BMMY and BSMM media. According to analysis (*p* < 0.05), the asterisk (*) indicates significant differences in HIP concentration between HF7 and CL4 in both media

**Fig. 10:**
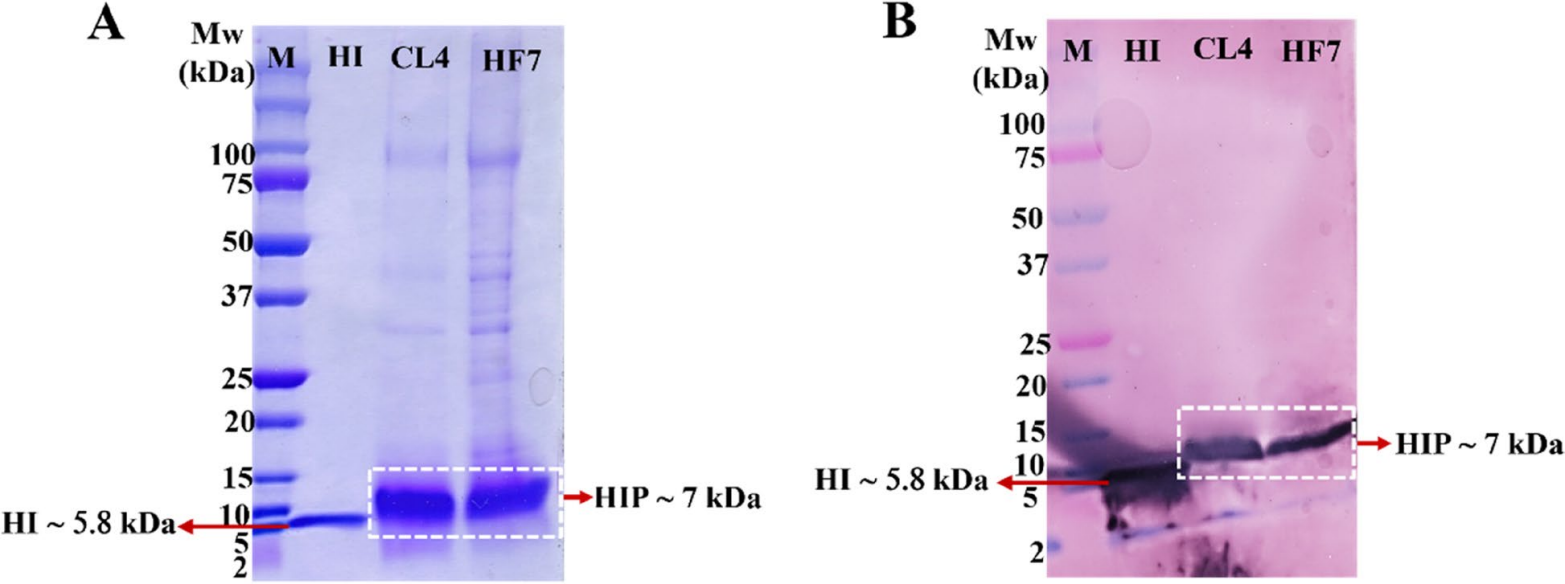
Western blot analysis. **A** SDS-PAGE of the HF7 and CL4-secreted protein showed the HIP. **B** The PVDF membrane after blotting. HF7 = HIP secreted from a full-length α-factor clone, CL4 = HIP secreted from a truncated α-factor clone, M = Protein marker, HI = USP human insulin standard

The HIP expressions of these selected clones were conducted on two types of media, BMMY and BSMM. The HIP protein analysis by SDS-PAGE of the culture supernatants after 72 h of methanol induction in both BMMY and BSM media shows that the HIP expression level of CL4 is higher than that of HF7 (Fig. 9A, B). ImageJ and statistical analysis were then performed on the SDS-PAGE results of the HI-USP standard, HIP expressed in BMMY, and HIP expressed in BSMM media results to quantify HIP expression in CL4 and HF7 clones (Fig. 9C). Two-way ANOVA shows a significant difference in the levels of HIP expression of CL4 and HF7 in both media (*p* < 0.05). This analysis showed that the HIP concentration of CL4 was significantly higher than that of HF7 (Fig. 9D). In addition to SDS-PAGE, Western blot analysis using an insulin/proinsulin monoclonal antibody recognizing human insulin precursor (~ 7 kDa) and human insulin (~ 5.8 kDa) confirmed that the protein secreted by HF7 and CL4 clones was a human insulin precursor with a size of ~ 7 kDa (Fig. 10).

### Full-length and truncated α-factor secretory protein structure prediction

**Fig. 11:**
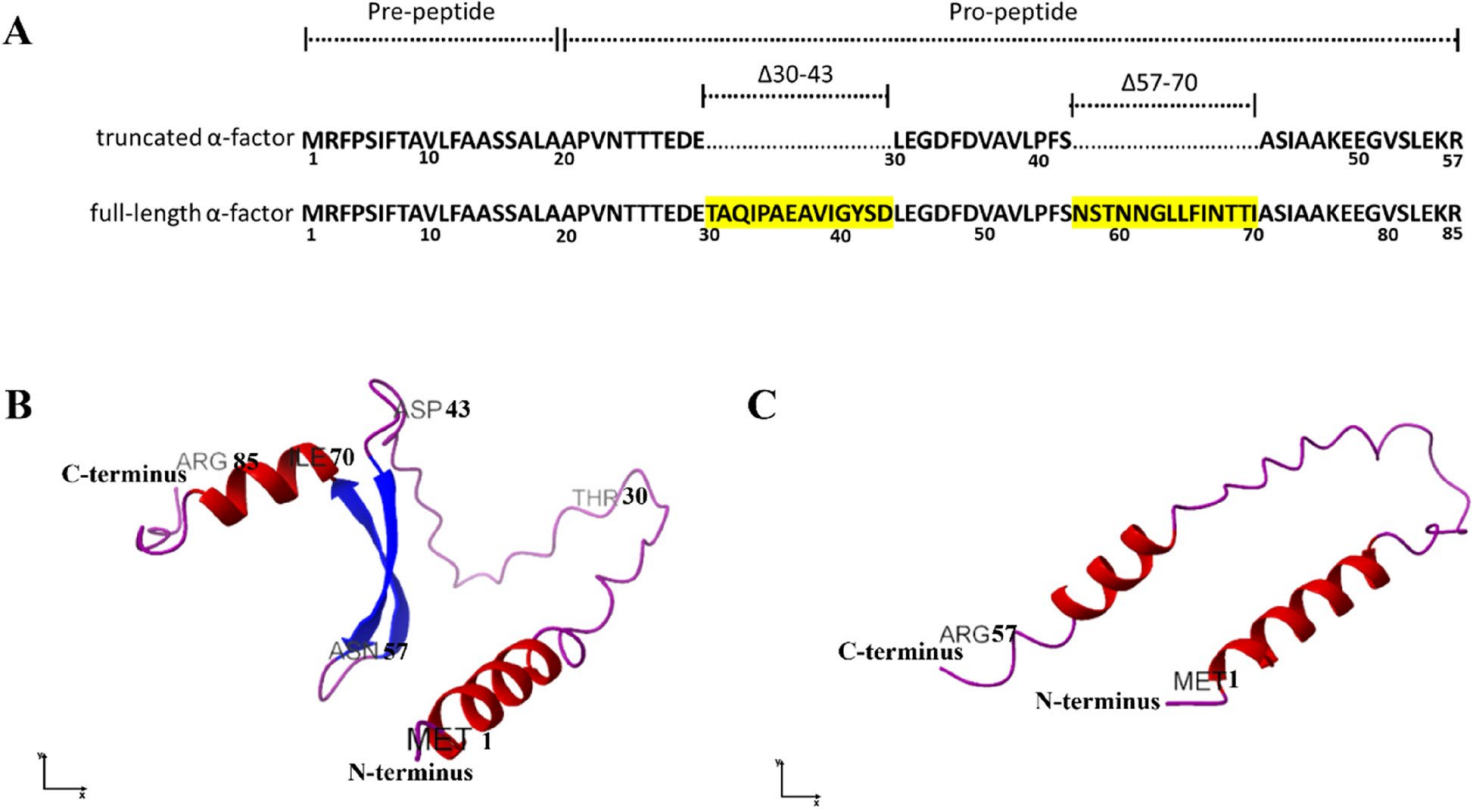
Protein structure prediction. **A** Schematic representation of full-length and truncated α-factor [deletion (yellow highlighted) in part Matα: Δ30-43 and Δ57-70]. **B** Predicted 3D model of full-length α-factor with 85 amino acids, and **C** truncated- α-factor with 57 amino acids

To predict the secondary structure of a full-length and truncated α-factor secretory signal protein, the AlphaFold program was used. Figure 11A shows the 85 amino acids constitute a full-length α-factor secretory signal sequence, which can be divided into two regions: pre-peptide (consisting of 19 amino acids) and pro-peptide (66 amino acids). Furthermore, the structure prediction of the full-length α-factor protein, visualized in Fig. 11B, showed that the protein has the α-helices, β-sheets, and loops structures. Specifically, amino acids “…FPSIFTAVLFAAS…” (number 3–15 in the pre-peptide region) and amino acids “… TTIASIAAK…” (number 68–76 in the pro-peptide region) are predicted to form an alpha-helical structure. Meanwhile, two beta-sheet structures are formed from the pro-peptide region, “… VAVLPFS…” and “… NGLLFIN…” (amino acids 50–56 and 61–67), further the loop structure connects the first alpha-helix and the first beta-sheet (amino acids 16–49), also connecting two beta-sheets (amino acids 57–60), and finaly the loop structure was formed by amino acids number 77–85.

On the other hand, Fig. 11C shows that the prediction structure of the truncated α-factor has two α-helices in the pre and pro-region connected with the loop structure. After deletion in part Matα: Δ30–43 and Δ57–70, where part Δ30–43 consists of amino acid sequences “…TAQIPAEAVIGYSD…” and part Δ57–70 with amino acids “…NSTNNGLLFINTTI…”. The truncated α-factor protein structure prediction showed that the β-sheet structures were lost, that is, in the full-length α-factor the predicted β-sheet structure is located in amino acids 50–56 and 61–67 (shown in Fig. 11B).

## Discussion

HF clone characterization consists of genetic validation, multi-copy identification (PTVA), best-expressing clone screening, and clone stability analysis, as previously described for CL4 clones [29, 31, 32]. Genetic validation by PCR in Fig. 3A showed that, as in CL4 clone, single crossover recombination occurred in all HF clones at the 5′-regions of the *AOX1* locus in their genome [29]. The bands of these PCR products also revealed that the nine HF clones were Mut^+^ (methanol utilization plus) phenotype which means that two genes of *AOX* (*AOX1* and *AOX*2) in the *P. pastoris* genome are intact and active [45]. Moreover, the multiple sequence alignment of nine HF clones confirms the belief that nine recombinant clones were inserted with pD902-IP-full-length α-factor cassette expression, as shown in Fig. 3B.

The PTVA method was conducted to identify putative recombinant strains containing multiple copies of the gene of interest to increase the titer of recombinant protein secretion [31, 34, 35, 46–48]. Previously, the HIP expression of the representatives of eight HF parental clones from the master plate (HF1, HF2, HF3, HF6, HF7, HF8, HF9, and HF10) had already been established [30]. The initial research by Sunga et al. in [34] reported that after the PTVA process, 40% of the selected clones showed a three to fivefold increase in vector copy number. In addition, Aw and Polizzi also reported that the GFP protein secretion yield was improved in the PTVA strains [35]. Based on this information, the PTVA plate for HF clones was performed, expecting to obtain a higher recombinant protein expression (Fig. 5). It has also been reported in some other studies that an increase in copy number could promote the increase of both intracellular [47, 49, 50] and extracellular protein titers [34, 35, 47, 48].

The stability of HIP expression of the best expressing clone, HF7 (shown in Fig. 6), was evaluated in five batches from generation 30.0 to 80.2 with 2% methanol induction without the presence of zeocin. Figure 7A shows that HIP was expressed only up to generation 55.6. However, genetic investigation by PCR using the *AOX1* primer in the HF7 genome from generation 30.0 to 80.2 shows that clone HF7 still retains the insulin expression cassette in the genome (shown in Fig. 7B). Compared to clone HF7, CL4 has a more stable HIP expression when grown without zeocin from generation 24.1 to 72.2 [32].

The *P. pastoris* X33 used in this study can utilize methanol as its sole carbon source and energy. This interesting property is supported by two genes in the *P. pastoris* genome, named *AOX1* and *AOX*2, which encode the alcohol oxidase enzyme [8, 51] that is involved in the methanol utilization pathway and are linked to the tightly regulated, strong methanol-inducible *AOX1* promoter [52]. This study found that expression level of HIP decreased with increasing methanol-induction concentrations (3, 4, and 5% compared to 2%), as shown in Fig. 8. This may be caused by methanol oxidation, which is also associated with the formation of hydrogen peroxide as a by-product which eventually leads to cell stress that could induce cell damage or death [53]. Another finding is that the Mut^+^ strains are sensitive to transient high methanol concentrations [53–56]. On the other hand, in our previous study, CL4 has an optimal methanol induction for the HIP expression of about 2–4% [57].

To confirm that deletion of the α-factor secretory leader (truncated version) could increase HIP secretion into the culture supernatant, similar to other reported proteins [25], the HIP expressions in two different clones of HF7 and CL4 in BMMY and BSMM media were compared. The HF7 and CL4 have full-length and truncated α-factor, on their expression cassette. The BMMY is a complex medium commonly used to allow better growth and mass accumulation of *P. pastoris*, especially for the expression of a secreted protein. It contained yeast extract and peptone as nutrient-rich substrates, which could help stabilize the secreted protein and reduce or prevent its proteolysis [33]. However, the use of BBMY as a medium could be economically challenging for large-scale recombinant protein production. As a result, large-scale production strategies use the chemically defined growth medium such as BSM due to its lower cost than a complex medium, simpler downstream purification processes, and in many cases, higher batch-to-batch consistency [58]. Therefore, in this study, the HIP expression in BMMY and BSMM media was compared.

The full-length α-factor secretory signal sequence has been used in other studies to secrete HIP in *P. pastoris* [13, 14, 16, 59]. However, attempts to enhance HIP secretion can still be made by using a modified α-factor secretory signal sequence. In some studies, *P. pastoris* has been known to fail to secrete some recombinant proteins efficiently even when using a proper secretion leader (reviewed by Puxbaum et al. [60]). Moreover, the pro-region in the α-factor consists mainly of hydrophobic amino acids, which are known to play an essential role in protein folding and slow down the rate of protein transport into the ER [23]. In the other study, Lin-Cereghino et al. [25] deleted the pro-region of the α-factor secretory signal sequence at Δ30–43 and Δ57–70, which resulted in HRP protein secretion in the mutant Matα: 30–43, 57–70 yeast, approximately 140% of the wild type. These reports are consistent with our findings that the CL4 clone, using a truncated α-factor, has approximately 8.97 times higher HIP protein secretion than the HF7 (full-length) in BMMY and 1.17 times higher in BSMM media (Fig. 7).

The AlphaFold program was run to predict the secondary structure of a full-length and truncated α-factor secretory signal protein. The most common secondary structural elements in proteins are α-helices, β-sheets, and random coils. Backbone interactions parallel to the helix’s primary axis form and maintain α-helices. These interactions are formed by hydrogen bonds between the carbonyl oxygen and amino nitrogen of the *i*th and *i* + 4th amino acids. The side chains of all residues in the α-helix are directed outwards and away from the helical axis, and the presence of polar or charged side chains in the helix can facilitate additional interactions with other side chains in the helix or with other elements outside of the helical structure, providing the necessary additional stability [45]. β-sheets have preferences for specific amino acids, particularly at their termini. Despite their weakness, these proclivities are sufficient to predict secondary structure better than chance [61].

The protein structure prediction of the α-factor in the pre-region (Fig. 9B) confirmed its prominent role and suggested that it forms an alpha helix that binds to signal recognition particles needed to enter the ER, as previously studied by Stern et al. [62]. Meanwhile, the pro-region plays a vital role in the membrane translocation of the recombinant protein. Lin-Cereghino et al. [25] hypothesized that deleting the hydrophilic amino acids in the pro-region should affect the secretion process of the recombinant protein. Deletion of Matα: Δ30–43 (hydrophilic amino acids) and Matα: Δ57–70 in the truncated α-factor resulted in the elimination of beta-sheets structure, as shown in Fig. 9C. Lin-Cereghino [25] reported that the deletion in parts Δ30–43 of the pro-region could increase secretion by approximately 20–30%, and removal of residues Matα: Δ57–70 increased secretion by at least 50%. The residues (Matα: Δ30–43 and Matα: Δ57–70) of the pro-region may constrain the interactions for proper secretion because the amino acids that make up the pro-region are thought to slow down the rate of transport to provide ideal folding conditions for the protein to be secreted. As a result, removal of the beta-sheet structures could release the loop (amino acids 44–56), allowing cargo proteins attached to it to travel more efficiently along the secretion pathway [63].

## Conclusions

The results of this study showed that the CL4 clone, which employed a truncated α-factor leader sequence with a deletion in parts of Matα: Δ30–43 and Matα: Δ57–70 in the HIP expression cassette *P. pastoris*, could secrete the HIP at a significantly higher level than the HF7 clone (a full-length α-factor leader sequence). These findings confirmed that the truncated α-factor (Matα: Δ30–43 and Δ57–70) could be used as an alternative secretory signal sequence for the higher expression of HIP compared to the full-length α-factor.

## Data Availability

All data generated or analyzed during this study are included in this published article.

## Abbreviations

HI: Human insulin
HIP: Human insulin precursor
FDA: Food and drug administration
*AOX1*: Alcohol oxidase 1
*PHO1*: Acid phosphatase 1
*SUC2*: Sucrose 2
Matα: Matting α-factor
Mut: Methanol utilization
*AOX1F*: Alcohol oxidase 1 forward
*AOX1R*: Alcohol Oxidase 1 reverse
PTVA: Post-transformational vector amplification
YPD: Yeast extract, peptone, d-glucose
BMGY: Buffered glycerol complex medium
BMMY: Buffered methanol complex medium
YNB: Yeast nitrogen base
WT: Wild-type
BSMM: Methanol basal salt medium
BSMG: Glycerol basal salt medium
